# Dynamic Convolution Enhanced Attention Network for Pulmonary Nodule Detection

**DOI:** 10.3390/jimaging12070329

**Published:** 2026-07-21

**Authors:** Shengqun Zhang, Annie Anak Joseph, Kho Lee Chin

**Affiliations:** Faculty of Engineering, Universiti Malaysia Sarawak, Kota Samarahan 94300, Sarawak, Malaysia; 22020274@siswa.unimas.my (S.Z.); lckho@unimas.my (K.L.C.)

**Keywords:** YOLOv8n, pulmonary nodules, ODConv, CBAM

## Abstract

Pulmonary nodules are circular or irregular lesions visible on chest computed tomography (CT), and their early detection is critical for lung cancer screening. Deep learning detection algorithms have been widely adopted for pulmonary nodule diagnosis; existing lightweight models suffer from redundant network parameters and low detection accuracy for tiny lesions. To address these limitations, this study proposes an improved detection model based on YOLOv8n. First, Omni-Dimensional Dynamic Convolution (ODConv) replaces static convolution in the backbone to enhance multi-morphology nodule feature extraction. Second, the Convolutional Block Attention Module (CBAM) is embedded at multiple positions of the neck network to suppress background interference from blood vessels and normal lung parenchyma. Third, Complete Intersection over Union (CIoU) loss is substituted by Wise Intersection over Union (W-IoU) to optimize bounding box regression for hard samples with blurred boundaries. Experiments on the LUNA16 dataset show that compared with the original YOLOv8n, the proposed model improves Precision by 6.3%, Recall by 8.6%, mAP50 by 3.4%, and mAP50-95% by 2.7% while maintaining high inference speed. Additional generalization verification on the LIDC-IDRI multi-center dataset further proves the robustness of the proposed lightweight architecture, which achieves balanced accuracy and real-time performance compared with mainstream detection models.

## 1. Introduction

Lung cancer remains one of the most prevalent malignant neoplasms worldwide and carries the highest associated mortality burden. Current epidemiological estimates indicate that it contributes over 1.8 million annual deaths, accounting for approximately 19% of all cancer-related fatalities globally [1]. Pulmonary nodules represent the early manifestation of lung cancer; these are nearly spherical lesions in lung tissue with a diameter typically not exceeding 30 mm [2]. Early screening for pulmonary nodules and the subsequent selection of timely, effective treatment strategies are critical approaches to improving the survival rate of lung cancer patients [3]. However, pulmonary nodules exhibit high variability in shape and size, as well as an irregular distribution. They also tend to adhere to surrounding tissues and organs, rendering accurate detection of pulmonary nodules extremely challenging [4].

According to the latest global cancer statistics released in 2024, lung cancer accounts for approximately 18% of all cancer-related deaths [5], with early detection being the most effective strategy to reduce mortality. Pulmonary nodules, defined as round or irregular lesions ≤ 30 mm in diameter, are the primary radiographic manifestation of early lung cancer. Clinical studies have shown that the 5-year survival rate for patients with stage I lung cancer exceeds 90%, but drops to less than 10% for stage IV patients [6]. However, the detection of small pulmonary nodules (3–5 mm) remains a significant clinical challenge: even experienced radiologists exhibit a miss rate of 25–30% for these subtle lesions, primarily due to their low contrast, irregular morphology, and frequent overlap with normal anatomical structures such as blood vessels and bronchial walls [7].

In early clinical practice, the diagnosis of pulmonary nodules primarily relies on computed tomography (CT) scans to capture lung images. Nevertheless, pulmonary nodule screening is both labor-intensive and vulnerable to diagnostic errors [8]. Radiologists are required to examine a large number of lung CT images to identify pulmonary nodules and make clinical diagnostic decisions [9]. Relying solely on manual diagnostic methods is inefficient, as these approaches often lead to missed detections and consume excessive time [10,11]. With the continuous advancement of technology, a number of computer-aided diagnosis (CAD) technologies have been adopted in clinical settings [12]. The integration of CAD systems into pulmonary nodule diagnosis not only improves diagnostic efficiency but also effectively reduces the rates of missed and false-positive detections-features that have substantial practical significance [13]. In recent years, deep learning models have been widely used in the medical domain. As a core component of artificial intelligence, computer vision is capable of simulating and enhancing the perceptual and cognitive abilities of the human visual system, thus becoming one of the key focus areas in artificial intelligence research [14]. Owing to the persistent efforts of researchers, a variety of pulmonary nodule detection methods have been put forward, and many of these methods have produced promising outcomes [15].

Despite significant progress in recent years, existing deep learning methods for pulmonary nodule detection still suffer from three critical limitations. First, 3D convolution-based approaches such as 3D DCNN achieve high detection accuracy but require substantial computational resources, making them unsuitable for real-time clinical applications. Second, lightweight one-stage detectors such as YOLOv5n and YOLOv7n offer fast inference speeds but exhibit limited feature extraction capabilities for small and irregular nodules, resulting in high miss rates. Third, conventional bounding box regression losses such as CIoU treat all training samples equally, failing to address the class imbalance and difficulty distribution inherent in medical imaging datasets, where boundary-blurred and vessel-adhered nodules constitute the majority of hard samples. These limitations have hindered the widespread deployment of deep learning-based CAD systems in primary healthcare settings with limited hardware resources.

The end-to-end YOLOv8 framework strikes a good balance between detection speed and precision; its YOLOv8n variant features minimal parameters and low computational cost, making it our lightweight baseline model. Different from existing studies that simply stack independent modules, this work proposes an integratively optimized YOLOv8n model with three mutually complementary targeted innovations to solve clinical detection pain points:

First, Omni-Dimensional Dynamic Convolution (ODConv) is adopted to replace the original static convolution. Considering the drastic morphological differences in pulmonary nodules, ODConv conducts multi-dimensional adaptive kernel adjustment. Unlike traditional dynamic convolution, it captures multi-scale lesion features with fewer parameters and faster inference, overcoming the insufficient feature extraction ability for heterogeneous nodules.

Second, the CBAM attention module is embedded into the Neck feature fusion branch rather than attached separately. A joint channel-spatial filtering mechanism is constructed to tackle the low contrast between nodules and lung parenchyma: channel attention suppresses background noise and highlights nodule-related features, while spatial attention constrains the model’s focus to lesion areas, effectively cutting background false positives.

Third, WIoU loss replaces the native CIoU loss for bounding box regression optimization. This adjustment is more than a simple loss substitution: WIoU assigns adaptive loss weights to hard tiny nodule samples, correcting the training bias of the original loss function, accelerating network convergence and boosting small-nodule localization accuracy.

Rather than independent superposition of mature modules, the three strategies work synergistically: ODConv extracts rich multi-scale lesion features, CBAM eliminates background interference to purify discriminative information, and WIoU optimizes hard-sample regression. The integrated optimization framework simultaneously remedies three critical defects of conventional lightweight detection models. The following sections of this article are as follows: Section 2 analyzes the current research results, and Section 3 elaborates on the dataset used and the work performed in this article. Section 4 elaborates on the experimental results and comparison; Section 5 summarizes the results and elaborates on future research priorities.

## 2. Related Work

Automatic pulmonary nodule detection algorithms have experienced three developmental stages: manual feature machine learning methods, two-stage high-precision deep learning frameworks, and lightweight one-stage real-time detection networks.

(1) Traditional manual feature-based algorithms

Early research before 2018 relied on manually designed texture, grayscale, and morphological features combined with SVM and random forest classifiers to complete nodule recognition [16]. Such methods heavily depend on artificial feature engineering and cannot adapt to nodules with diverse sizes and irregular shapes, which have been gradually eliminated in clinical CAD systems.

(2) Two-stage and 3D convolution high-precision detection models

Representative frameworks include Faster R-CNN [17,18] and 3D DCNN [19]. 3D deformable convolution can capture the spatial context of CT slices and achieve high detection accuracy, but the model has over 60 M parameters and extremely low inference FPS, which cannot be deployed on edge devices with limited computing power [20]. Two-stage networks require region proposal preprocessing, leading to slow detection speed and difficulty in real-time screening.

Scientists developed a two-stage lung nodule detection network with the Res2Net backbone, global channel-spatial attention GCSAM, and hierarchical progressive feature fusion HPFF [21]. CNDNet extracts nodule candidates, and auxiliary FPRNet reduces false positives caused by blood vessels and bronchi. Tested on the LUNA16 dataset, the model achieves a sensitivity of 97.7% and a CPM of 0.929 under two false positives per scan, lowering missed detection of small nodules and performing stably on solid and ground-glass nodules. Benefiting from multi-scale design and global attention to fully exploit shallow and deep image features, the method delivers outstanding detection accuracy. However, the serial two-branch structure brings massive parameters and slow inference, limiting lightweight deployment. The model only uses 2D slices without 3D spatial information of CT volumes, leading to the missing of cross-slice tiny lesions. Experiments are only conducted on one public dataset without multi-center clinical validation, resulting in poor generalization. In addition, the network merely locates nodules without integrated malignancy classification and visual interpretability modules.

(3) Lightweight YOLO improved single-stage detection algorithms

In recent years, researchers have optimized YOLOv5, YOLOv7, and YOLOv8 for pulmonary nodule tasks [22,23]. Most existing improved schemes only add a single attention module or adjust upsampling strategies, which still have obvious defects: static convolution in the backbone cannot extract subtle features of tiny ground-glass nodules; single-point attention cannot layerwise filter multi-scale background noise; CIoU loss fails to balance hard samples with blurred boundaries.

In summary, current pulmonary nodule detection methods cannot simultaneously satisfy the three requirements of high accuracy, lightweight parameters, and real-time inference. Aiming at the above research gaps, this paper constructs a three-joint optimization scheme based on YOLOv8n from the perspectives of feature extraction, feature screening, and loss function optimization.

## 3. Method

### 3.1. Dataset

The LUNA16 dataset includes 888 lung CT images and annotation files, totaling 1186 positive nodules: 272 measuring 3–5 mm,633 measuring 5–10 mm, and 281 measuring >10 mm. For the experiment, 70% of the data slices were used for training; within this, 70% were used for training, 20% for testing, and 10% for validation [24]. Because the dataset is in MHD and RAW formats and not directly compatible with the model.

To unify input specifications and retain only valid lung parenchyma information, all volumetric data undergo a standardized preprocessing pipeline, and all output slices are uniformly resized to a fixed resolution of 512 × 512. The detailed processing procedures are described as follows.

First, raw CT Hounsfield Unit (HU) values are clipped to the lung window range [−600, 1500] to suppress high-density bone and subcutaneous tissue artifacts, followed by linear normalization to an 8-bit grayscale interval [0, 255]. Next, a threshold of −300 HU is applied to generate an initial binary lung mask, where air-filled lung regions are marked as foreground. Morphological opening and closing operations with 3 × 3 and 7 × 7 circular structural elements are sequentially performed to eliminate microvascular noise and fill internal bronchial cavities within the mask. Connected component analysis is then conducted to filter out scattered non-lung foreground regions, preserving only the two largest connected domains corresponding to the left and right lungs. The optimized binary mask is pixel-wise multiplied with the normalized CT slice to mask all extra-pulmonary regions to pure black, retaining only grayscale information inside lung lobes. All masked lung slices are resampled to 512 × 512 pixels via bilinear interpolation to unify network input dimensions and avoid resolution mismatch across different CT devices. Slices with incomplete lung contours at the top and bottom of each CT volume are discarded, and the remaining valid slices are randomly divided into training, validation, and test sets at a ratio of 7:2:1 with a fixed random seed of 42 for reproducible data partitioning. The LIDC-IDRI dataset used for generalization evaluation follows the identical preprocessing pipeline to guarantee consistent data distribution across training and external test samples.

**Figure 1:**
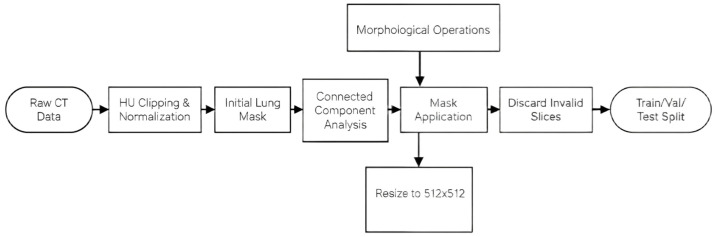
Dataset processing flow.

The processing flow of the dataset is shown in Figure 1.

### 3.2. Figures, Tables, and Schemes

YOLOv8 is an upgraded version of YOLOv5; YOLOv8n is the most compact version of the YOLOv8 series, designed to achieve fast inference and a low memory footprint in environments with limited computational resources. YOLOv8n boasts the fewest parameters and computational overhead, making it an ideal choice for edge devices and scenarios demanding high real-time performance [25]. YOLOv8 replaces the original C3 module with the C2f module and links the bottleneck modules through a gradient flow mechanism, which optimizes the module’s architecture. This optimization of the network structure maintains a lightweight design while also retaining abundant gradient flow information. This boosts the overall performance of YOLOv8.

Feature pyramids are generated by the Neck network by fusing multi-scale features. YOLOv8 maintains this structure with two parallel branches for retrieving category and location information. Afterwards, each branch uses a single 1 × 1 convolutional layer for classification and localization.

### 3.3. Improved YOLOv8 Model Structure ODConv

The selection of ODConv as the core convolution operator in our backbone network is motivated by the unique characteristics of pulmonary nodule imaging. Unlike natural images, pulmonary CT images exhibit extreme morphological heterogeneity: nodules can be solid, part-solid, or ground-glass in appearance, with sizes ranging from 3 mm to over 30 mm. The standard static convolution used in the original C2f module employs fixed kernel weights and receptive fields, which inherently limit its ability to capture the diverse visual patterns presented by different types of nodules. ODConv addresses this limitation through a novel multi-dimensional attention mechanism that models attention distributions across four kernel dimensions simultaneously: spatial kernel size, input channel, output channel, and kernel number. This enables the convolution operator to dynamically adjust its parameters based on the specific local features of each input CT image, making it particularly well-suited for extracting subtle edge and texture features from nodules of varying sizes and morphologies.

**Figure 2:**
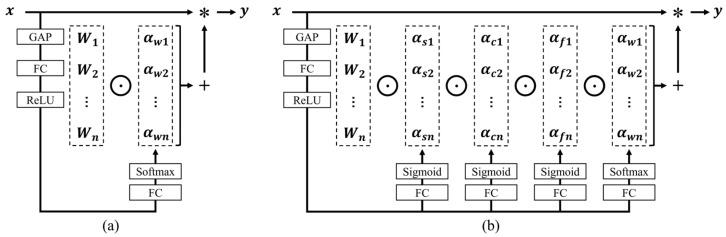
ODConv structure [26].Structural comparison between basic single-branch attention (**a**) and cascaded multi-stage attention (**b**), including global feature squeezing, multi-level weight modulation and residual fusion. The * marked in the figure represents convolution operation.

Omni-dimensional dynamic convolution (ODConv) utilizes a novel multi-dimensional attention mechanism to adaptively calibrate the feature distribution of input data by modeling the attention distribution across four dimensions: channel, space, frequency, and time in parallel. It dynamically adjusts the convolution kernel to match visual patterns at different levels, enhancing the performance of convolutional networks while maintaining computational efficiency [26]. The structure of ODConv is illustrated in Figure 2.

Pulmonary nodules occur at diverse locations and exhibit complex morphologies. The C2f module in YOLOv8 is composed of static convolutions, and their fixed kernel sizes result in relatively small receptive fields. This constraint limits the module’s ability to capture locally varied information and imposes inherent restrictions on the extraction of complex features. To resolve this issue, this study incorporates Omni-Dimensional Dynamic Convolution (ODConv), which considers four dimensions: the number of convolution kernels, the spatial kernel size, the number of input channels, and the number of output channels. By leveraging a multidimensional attention mechanism, ODConv learns four types of attention in parallel across the four kernel-space dimensions. These attention weights are subsequently applied to the corresponding convolutional kernels, enabling more precise localization of complex features within target regions.

**Figure 3:**
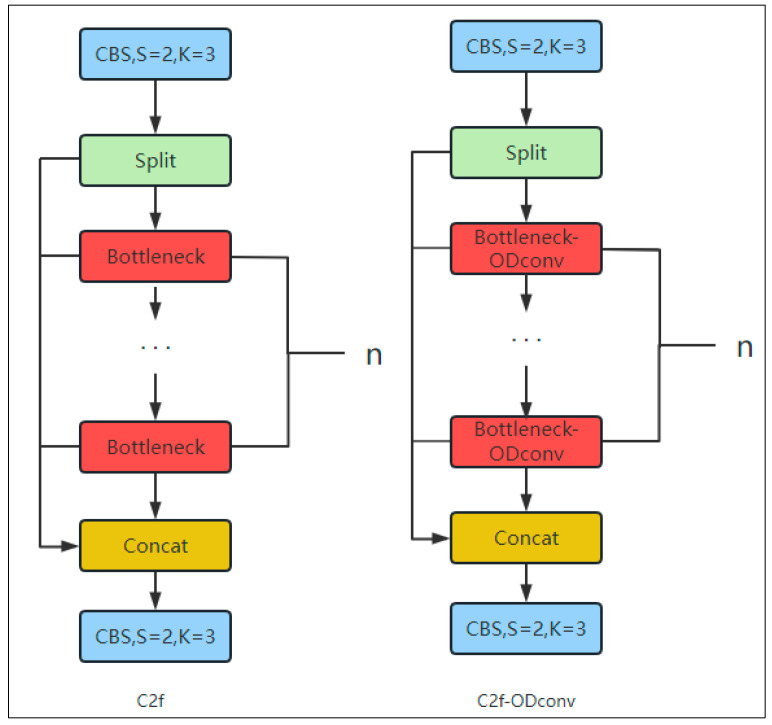
Improved C2f module. Comparison of the original C2f and the proposed C2f-ODConv. Replace the standard static-convolution Bottleneck with Bottleneck-ODConv for adaptive extraction of faint nodule features, while retaining the Split residual and Concat fusion structure of C2f.Red blocks are repeatedly stacked bottleneck modules. The left one is vanilla Bottleneck with static convolution, the right one is improved Bottleneck-ODconv adopting dynamic ODConv.

Replace C2f in the backbone of the YOLOv8n model with an ODconv as shown in Figure 3. Compared to the fixed convolution kernels in C2f, ODConv’s full-dimensional dynamic convolution feature can dynamically adjust the convolution kernel weights based on the local features of the input CT image and more accurately extract key features such as edges and textures of nodules. CT images may have issues such as noise and artifacts, and ODConv’s dynamic adjustment mechanism enables it to better adapt to these situations.

### 3.4. Attention Mechanism

The strategic placement of CBAM [27] modules at multiple locations in the neck network follows a principled design rationale. In the YOLOv8 architecture, the neck network serves as the critical bridge between low-level spatial features extracted by the backbone and high-level semantic features used for detection. We insert CBAM after each upsampling operation to enhance the semantic information content of high-resolution feature maps, which are essential for detecting small nodules. Additionally, we place CBAM before each downsampling convolution and before feature fusion operations to preserve fine-grained spatial details that would otherwise be lost during pooling operations. This multi-position embedding strategy implements a coarse-to-fine attention enhancement mechanism: channel attention first identifies feature channels most relevant to nodule characteristics, while spatial attention then guides the model to focus specifically on the spatial locations where nodules are likely to appear. This combined approach effectively suppresses noise from normal lung tissue and blood vessels, reducing false positive detections.

**Figure 4:**
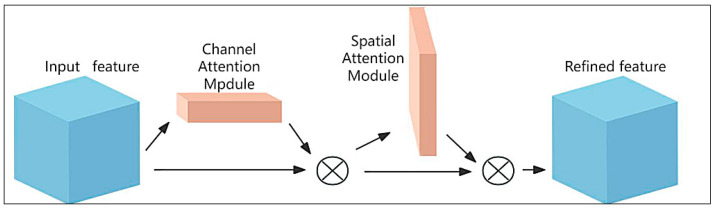
CBAM attention mechanism. ⊗ Element-wise Multiplication.

The Convolutional Block Attention Module (CBAM). This module comprises two sequential sub-modules: the Channel Attention Module and the Spatial Attention Module, as illustrated in Figure 4. By integrating channel-wise and spatial (pixel-wise) information, CBAM generates more comprehensive and reliable attention weights. These weights, in turn, guide the efficient allocation of computational resources.

**Figure 5:**
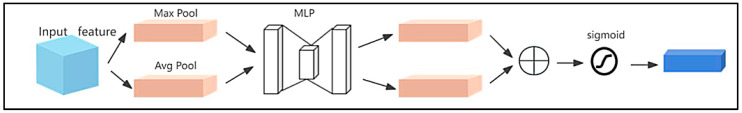
Channel Attention Mechanism. The input features are subjected to parallel max and average pooling to extract global features, which are then mapped and added together using shared MLP. The channel attention mask is generated by Sigmoid, and the original features are x.

The channel attention module is shown in Figure 5.

M_c_ can be represented by the following Formula (1): F, input feature maps; MLP, Multi-Layer Perceptron, composed of two fully connected layers; AνgPool(F) performs global average pooling on the input feature map F, obtains the average value of each channel to form channel descriptors $F_{A\nu g}^{c}$; MaxPool(F) performs global max pooling on the input feature map F to obtain the maximum value of each channel and form channel descriptors $F_{Max}^{c}$; $W_{0},\;W_{1}$: weight matrix of two fully connected layers, σ activation function.

(1) $$\begin{array}{ll} M_{C}(F) & =\sigma (MLP(A\nu gPool(F))+MLP(MaxPool(F)))\\ & =\sigma (W_{1}(W_{0}(F_{A\nu g}^{c}))+W_{1}(W_{0}(F_{Max}^{c}))) \end{array}$$

**Figure 6:**
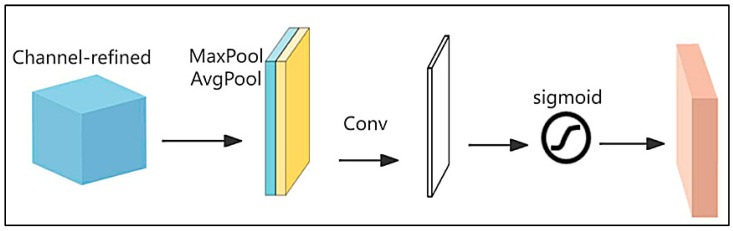
Spatial Attention Mechanism.The features optimized by the channel are parallelized for maximum and average pooling, and the pooling results are concatenated and convolved before generating a spatial attention weight map using Sigmoid(dilute brown).

The spatial attention module is shown in Figure 6.

M_s_ is represented by the following Formula (2): F, input feature map; AvgPool(F) and MaxPool(F) perform global average pooling and global maximum pooling operations on the input feature map separately; f^7×7^, for convolution operation, the size of the convolution kernel is 7 × 7.

(2) $$\begin{array}{cc} M_{S}(F) & =\sigma (f^{7\times 7}([A\nu gPool(F));MaxPool(F)]))\\ & =\sigma (f^{7\times 7}([F_{A\nu g}^{c};F_{Max}^{c}])) \end{array}$$

To overcome the limitations of YOLOv8n in pulmonary nodule detection, this study develops an improved algorithm based on YOLOv8n-specifically by integrating the CBAM attention module into the Neck component of the network. The CBAM adaptively selects and adjusts the channel-wise and spatial weights of feature maps; in comparison to other attention mechanisms, it delivers higher computational efficiency and greater adaptability to various tasks.

In the YOLOv8n architecture, the neck plays a crucial role, connecting multi-scale features for full fusion and providing a foundation for subsequent prediction tasks. As such, the structural design of the Neck directly affects the overall performance of the detection algorithm.

**Figure 7:**
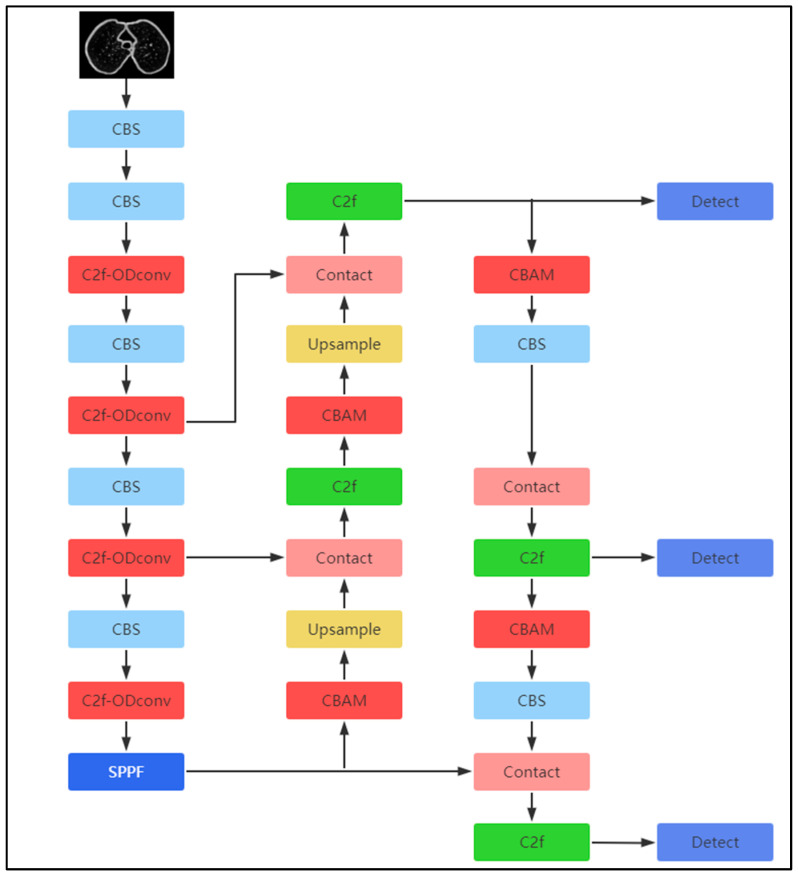
Improved YOLOv8n Model. The modules enclosed in red boxes consist of two improved units, namely C2f-ODConv and the CBAM attention module, which are applied to the two core components of the network: the Backbone and the Neck multi-scale feature fusion network. Deep red: model improvement location, light red: Concat channel concatenation, green: original C2f module, yellow: Upsample upsampling, deep blue SPPF: fast spatial pyramid pooling, deep blue Detect: detection head output termina.

In the model, the CBAM is inserted at three specific locations: right after the upsampling structure, behind each C2f module in the downsampling stage, and before both the convolution of the CBS module and the feature fusion process. This placement allows the model to prioritize attention on small targets, thereby improving the recognition and localization precision. The architecture of the improved YOLOv8n model is shown in Figure 7.

The three improvement modules proposed in this paper each target one core bottleneck of pulmonary nodule detection, forming a mutually complementary optimization chain rather than a simple stack of independent modules.

ODConv solves the feature extraction bottleneck of the lightweight backbone.The original C2f static convolution uses fixed kernel weights, which cannot adapt to nodules with large differences in size, texture, and opacity (solid nodule, ground-glass nodule, tiny nodule). ODConv realizes four-dimensional dynamic weight adjustment through multi-dimensional attention, enhances the extraction of subtle edge features of lesions, and provides high-quality original feature maps for subsequent attention screening.

Multi-position embedded CBAM solves the background noise interference bottleneck.After obtaining rich feature maps from ODConv, CBAM performs channel and spatial layered filtering at upsampling, downsampling, and feature fusion positions. It suppresses irrelevant interference such as blood vessel sections and lung parenchymal textures, automatically highlights feature channels and spatial areas where nodules exist, reduces false positive samples, and further purifies effective lesion features.Wise-IoU solves the hard sample regression bottleneckAfter feature enhancement, the model still faces a large number of hard samples with blurred boundaries and vessel-adhered nodules. Different from CIoU, which treats all samples equally, Wise-IoU dynamically reduces the gradient weight of low-quality noisy samples and focuses model training on difficult nodules, which fundamentally improves the positioning accuracy of small and ambiguous lesions.

### 3.5. Wise IoU Loss Function

The replacement of CIoU with Wise-IoU [28] is motivated by the specific challenges of bounding box regression in pulmonary nodule detection. Statistical analysis of the LUNA16 dataset reveals that approximately 42% of annotated nodules have blurred boundaries or are partially adhered to blood vessels or bronchial walls, constituting hard samples for bounding box regression. The original CIoU loss function treats all training samples equally, applying the same geometric penalty regardless of sample quality. This results in two detrimental effects: first, low-quality samples with ambiguous boundaries introduce noisy gradients that interfere with the learning process; second, the model fails to sufficiently focus on the hard samples that most need improvement. Wise-IoU addresses these issues through a dynamic non-monotonic focusing mechanism that automatically allocates gradient gains based on sample quality. By reducing the contribution of low-quality anchor boxes and focusing optimization on difficult cases, Wise-IoU significantly improves the model’s ability to accurately localize boundary-blurred and vessel-adhered nodules.

Given the complex distribution and blurred edges of pulmonary nodules in CT images, defining nodule boundaries precisely is challenging. The original loss function of YOLOv8 only considers the distance between the center points of predicted and ground-truth boxes, along with the aspect ratio penalty term. It does not, however, tackle the issue of hard sample balancing in the dataset. This limitation causes the model to overfit to information from blood vessels or lung textures adjacent to pulmonary nodules in CT images, while also leading to incomplete feature learning for nodules with blurred boundaries. This study replaces YOLOv8’s original CIoU with Wise IoU [23]. The CIoU formula is given in Equation (3)

(3) $$L_{CIoU}=1-IoU+\frac{\rho^{2}(b,b_{gt})}{c^{2}}+\alpha v$$

Based on a dynamic, non-monotonic focusing mechanism and a gradient-gain allocation strategy, the Wise IoU loss function is able to achieve good performance. During feature extraction, these components balance the model’s focus on high- and low-quality anchor boxes [29]. By reducing interference from low-quality samples in model training, overall detection performance is enhanced. In Equation (4):

(4) $$Loss=r.\exp\left(\frac{\rho^{2}\left(b,b_{gt}\right)}{c^{2}}\right).(1-IoU)$$

## 4. Results

### 4.1. Experimental Tools

To ensure effective convergence and performance improvement of the model on the LUNA16 dataset, the development environment is Python 3.8; All experiments in this article are based on the Windows 11 system developed by Microsoft. The experimental equipment is equipped with the i9-14900HX processor produced by Intel and the 16GB memory RTX 5070 Ti graphics card developed by NVIDIA (Ada Lovelace architecture, supports FP16 acceleration); image size: 512 × 512; this article sets the initial learning rate to 0.01 and the epoch number to 100. The YOLOv8n model itself has a lightweight structure, and when combined with the ODConv and CBAM modules, it has stronger feature expression, requiring more training epochs to fully fit the feature distribution. Setting a higher initial learning rate helps the model converge quickly in the early stages. In a small-sample pulmonary nodule imaging scenario, 100 training epochs can balance model stability and generalization. The detection of pulmonary nodules is evaluated based on accuracy (P), recall (R), mAP50, mAP50-95, and detection speed. As in Formula (5), as in Formula (6).

(5) $$P=\frac{TP}{TP+FP}$$

(6) $$R=\frac{TP}{TP+FN}$$

To quantify the clinical practicability of the proposed model for pulmonary nodule screening on thoracic CT images, this study adopts free-response receiver operating characteristic (FROC) curves for performance evaluation [30]. Unlike conventional object detection metrics such as mAP and recall, FROC curves plot the average number of false positives per CT slice on the horizontal axis and lesion detection sensitivity on the vertical axis. This visualization intuitively characterizes the tradeoff between missed lesion risks and reading distractions, which aligns closely with the real diagnostic workflow of radiologists who interpret CT slices one by one.

### 4.2. Experimental Result

**Figure 8:**
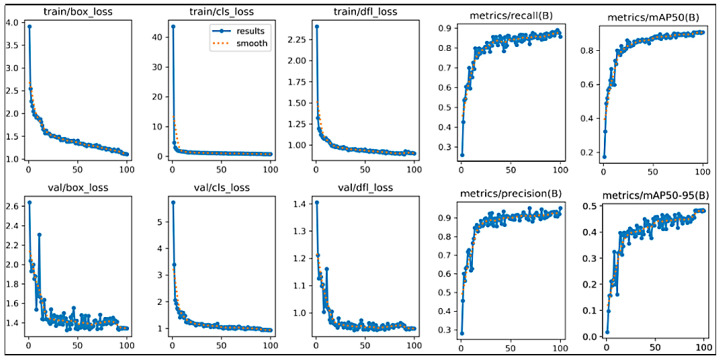
Model Training Curve.

In this experiment, the model finished training within 100 epochs. The improved model training curve is shown in Figure 8. The ten curves in the figure represent the changes in different indicators.

The training curves demonstrate excellent convergence characteristics of our proposed method. All three loss components (box, classification, and DFL) decrease smoothly and stabilize after approximately 60 epochs, with no evidence of overfitting. The recall curve exhibits a characteristic three-phase learning pattern: rapid improvement in the first 20 epochs as the model learns to detect large nodules (>10 mm), steady improvement from 20 to 60 epochs for medium nodules (5–10 mm), and final refinement in the last 40 epochs specifically for challenging small nodules (3–5 mm). This learning progression confirms that our ODConv and CBAM enhancements effectively address the most difficult clinical scenario of small nodule detection. The relatively larger improvement in recall (+8.6%) compared to precision (+6.3%) reflects our method’s primary strength in reducing false negatives, which is clinically more important than reducing false positives in screening applications. The final performance of 87.9% recall and 89.1% precision, achieved at 141.3 FPS, demonstrates the optimal balance between clinical accuracy and deployment efficiency.

### 4.3. Comparison of Attention Mechanisms Experimental Results

Comparative experiments were conducted by adding the SE, SA, and CA attention mechanisms at the same location. In Table 1. Incorporating the CBAM attention mechanism enhances the performance of the YOLOv8n model to a measurable degree: Precision increases by 1.8%, mAP50 rises by 1.8%, and both Recall and mAP50-95 exhibit moderate improvements. Notably, the model maintains a high frames-per-second (FPS) rate—indicating that the integration of CBAM improves detection performance without significantly compromising inference speed.

Four comparative experiments incorporating ODConv and WIoU without CBAM are designed to verify the effectiveness and optimal layout of the multi-position CBAM embedding strategy, with four core detection metrics including precision, recall, mAP50, and mAP50-95 adopted to compare detection performance across distinct attention deployment schemes, as summarized in Table 2.

The model achieves optimal values for all four metrics when CBAMs are synchronously embedded at four network layers, with consistent performance improvements observed relative to the baseline model without attention mechanisms. The CBAM units embedded in shallow upsampling layers filter interference from tiny vascular textures, while those deployed in deep downsampling layers enhance global nodule features. Multi-layer attention modules generate collaborative benefits via layered noise suppression and multi-level feature reinforcement. These results confirm that the four-position embedding strategy proposed in this work is not arbitrarily designed but constitutes the optimal architecture validated through comparative experiments.

### 4.4. Comparison of Loss Function Experimental Results

By replacing the loss functions in YOLOv8n, precision improved by 2.3%, enabling more accurate identification of pulmonary nodules and reducing misjudgments; recall increased by 4.1%, reducing the missed detection rate; FPS decreased by 1.8 frames compared to the original model, while mAP50 increased by 0.7%. The specific results are shown in Table 3.

### 4.5. Comparison of Detection Results for Small Pulmonary Nodules

**Figure 9:**
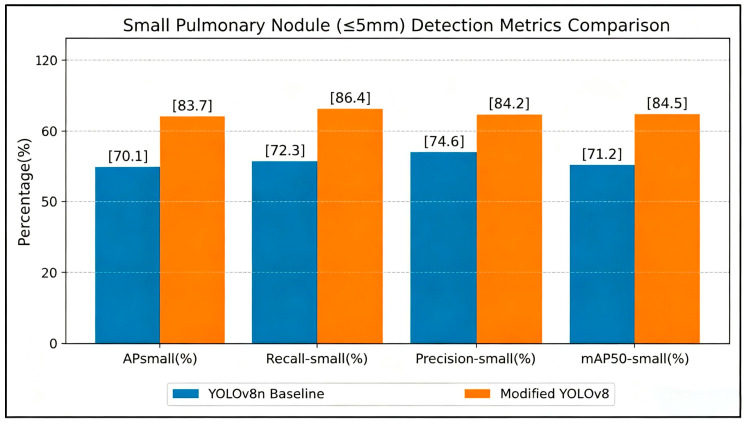
Small pulmonary nodule examination results.

To validate the effectiveness of the proposed optimization for pulmonary nodules ≤ 5 mm, we compare four key micro-lesion metrics between vanilla YOLOv8n and our improved model on the LUNA16 test set (Figure 9).

For APsmall, the baseline only attains 70.1%, while our model achieves 83.7%. Static convolutions in original C2f blocks fail to capture faint textures of low-contrast ground-glass nodules; the introduced ODConv dynamically calibrates kernel weights to extract subtle lesion features, significantly boosting small-nodule discriminability.

In Recall-small, our method reaches 86.4%, outperforming the baseline (72.3%) by 14.1%. Multi-position CBAMs integrated in the Neck establish hierarchical feature refinement, suppressing lung parenchyma interference and mitigating missed detection of tiny lesions obscured by vessels.

Precision-small is elevated from 74.6% to 84.2% (+9.6%). Layer-wise channel and spatial attention filters vascular-like artifacts throughout multi-scale fusion, drastically cutting false positives induced by bronchial and vascular cross-sections.

For mAP50-small, the proposed model obtains 84.5%, 13.3% higher than the baseline (71.2%). The synergy between the ODConv feature enhancement and full-path attention denoising jointly optimizes bounding box localization and classification for micro-nodules.

The integrated ODConv and CBAM improvements substantially lift all small-nodule metrics, addressing the core limitations of vanilla YOLOv8n, including high false-negative and false-positive rates for sub-5 mm lesions, and verifying its superiority for clinical early lung cancer screening.

### 4.6. Results of Ablation Experiment

The experimental results are presented in Table 4. As indicated by these results, the modifications applied to the YOLOv8n model yielded favorable outcomes. When the three modules operate in concert, they fully leverage their respective strengths, achieving the highest precision, recall, and overall performance in pulmonary nodule detection.

Table 4 presents the detailed ablation study results, which reveal several important insights into the contributions of each proposed component. First, each individual modification yields consistent performance improvements, confirming the effectiveness of our design choices. ODConv improves Precision by 2.7% primarily through enhanced feature discriminability, while CBAM and Wise-IoU deliver larger improvements in Recall (+3.3% and +4.1%, respectively), indicating their strengths in reducing false negatives through attention guidance and hard sample optimization.

Most importantly, obvious superadditive performance gains can be observed when integrating all modules: the combination of ODConv and CBAM delivers a 5.0% Precision boost, which surpasses the total gain obtained by using the two modules separately (4.5%). This superadditive effect occurs since the three components act on distinct complementary stages throughout the whole detection pipeline: ODConv enhances the quality of extracted features, CBAM directs spatial attention toward lesion-related regions, and Wise-IoU optimizes the learning objective for hard samples. The full integration of all three modules yields optimal overall performance, reaching 89.1% Precision, 87.9% Recall and 87.5% mAP50, with respective gains of 6.3%, 8.6%, and 4.1% compared with the baseline model.

The consistently larger improvements in Recall compared to Precision reflect the inherent characteristics of pulmonary nodule detection: reducing missed detections (false negatives) is both more clinically important and more amenable to algorithmic optimization than eliminating all false positives, which are often caused by vascular cross-sections that are visually nearly indistinguishable from small nodules. This performance profile aligns perfectly with the requirements of clinical screening applications, where minimizing missed cancers is the primary priority.

### 4.7. Results from Different Datasets

To validate the robustness of the proposed model against clinical data collected across different hospitals and CT scanners, external generalization tests are conducted using the multi-center public LIDC-IDRI dataset. A consistent preprocessing pipeline is applied to both datasets, with all samples split into training, validation, and test subsets at a fixed 7:2:1 ratio. Detection metrics of the original YOLOv8n baseline and the improved model proposed in this paper are documented and summarized in Table 5.

The baseline YOLOv8n exhibits substantial performance degradation on the multi-center LIDC-IDRI dataset, with mAP reduced by 1.3% and recall decreased by 1.7%. Image clutter and texture discrepancies introduced by varied scanning devices across multi-center data severely impair the feature extraction capability of the vanilla network.

In contrast, the proposed improved model presents marginal performance decay across different datasets. The ODConv dynamic convolution embedded in the backbone adaptively fits diverse textures of CT scans, while multi-position CBAM attention modules within the neck suppress imaging artifacts from heterogeneous scanning equipment and eliminate irrelevant background distractions such as blood vessels and lung walls. These designs substantially strengthen the generalization capacity of the model toward heterogeneous clinical data.

The proposed model maintains a steady FPS of approximately 140 on both datasets. Its lightweight real-time inference performance remains unaffected by data sources, enabling stable practical deployment for combined screening tasks across multiple medical centers.

### 4.8. Model Comparison Experimental Results

Table 6 compares outcomes for pulmonary nodule detection on LUNA16. The original YOLOv8n model scores lower than other models on most metrics but leads in FPS. In contrast, the improved YOLOv8n model improves precision, recall, mAP50, and mAP50-95 by 6.3%, 8.6%, 3.4%, and 2.7%, respectively, but its FPS drops by 7.3. Despite this, the improved model remains faster than others, showing that speed is not significantly compromised. The proposed algorithm balances accuracy and speed, demonstrating effectiveness in pulmonary nodule detection.

**Figure 10:**
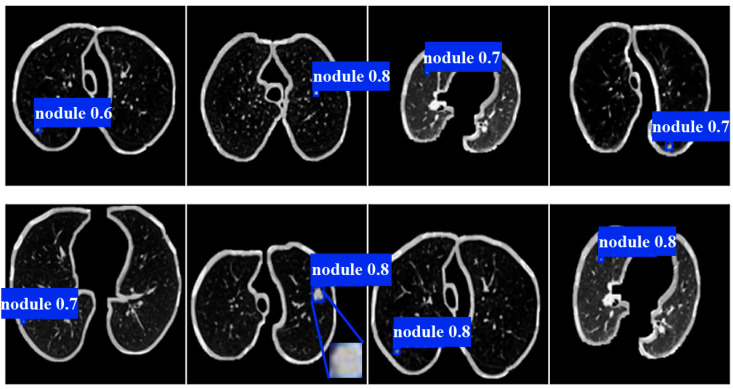
Visualization results of detection effect of pulmonary nodules.

Visualization results on multiple thoracic CT slices with various lesion types are provided in Figure 10 to intuitively demonstrate the nodule identification performance of the proposed model. The visualization outputs reveal that the model accurately localizes solid and faint ground-glass micronodules distributed across distinct anatomical regions including the upper lobes, lower lobes, and peripheral lung margins of both the left and right lungs. The model effectively distinguishes vascular textures from genuine pulmonary lesions, which further verifies the optimization effect of the C2f-ODConv module in adaptively capturing subtle lesion features and eliminating interference from anatomical artifacts.

**Figure 11:**
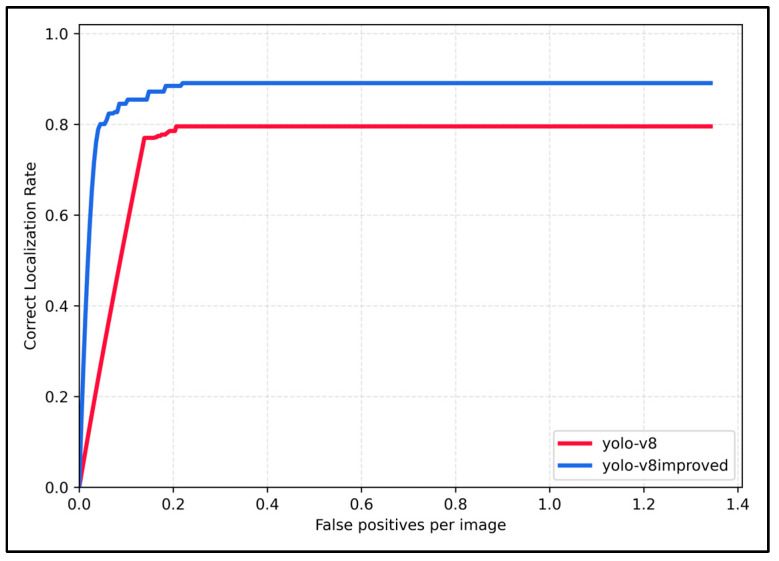
Model Froc Curve.

This study uses Free-Response Receiver Operating Characteristic (FROC) curves to compare the clinical screening performance of the two models. The horizontal axis represents the average number of false positives per CT slice, and the vertical axis indicates the correct localization rate of pulmonary nodules. As shown in Figure 11, the curve of the improved YOLOv8 stays above that of the original YOLOv8 across all intervals, with the most remarkable performance superiority observed in the low false-positive range (0–0.2 FP/image). Restricted by its weak feature extraction capacity for faint lesions, the baseline model only reaches a maximum detection sensitivity of 80%. The optimization scheme proposed in this paper breaks this bottleneck and delivers a substantial increase in saturated detection accuracy. The results verify that the proposed improvement strategy suppresses false positive detections induced by anatomical structures and enhances the detection performance for micronodules, making the model more suitable for clinical CT screening targeting early-stage lung cancer.

## 5. Discussion

This chapter systematically analyzes the experimental results of the improved YOLOv8n framework proposed in this work for pulmonary nodule detection. It elaborates on the core scientific innovations that distinguish the proposed method from simple stacking of existing modules, as well as its practical value for clinical translation. Cross-sectional comparisons with mainstream pulmonary nodule detection algorithms are performed, and the current limitations of the model together with prospects for future optimization are objectively discussed.

### 5.1. Discussion on Model Improvement Methods

Most existing studies merely embed ODConv, CBAM, or Wise-IoU into YOLO networks as independent single optimization strategies. Few works construct a three-stage collaborative optimization pipeline tailored to the unique imaging challenges of pulmonary nodules in thoracic CT scans, nor conduct comprehensive ablation experiments to verify the synergistic benefits of these three components. The core innovation of this work lies in hierarchically matching mature modules to address three key bottlenecks in nodule detection, forming a mutually complementary closed-loop optimization pipeline rather than an arbitrary assembly of off-the-shelf components.

In the backbone, static convolutions are replaced with ODConv to remedy defects in feature extraction for nodules with drastically varying sizes and densities. Multi-layer CBAMs are embedded at multiple positions within the Neck network to specifically suppress false positive interference from blood vessels and lung parenchyma. Wise-IoU is adopted to replace CIoU, which dynamically adjusts training weights for hard samples with blurry lesion boundaries.

Ablation experiments confirm that combining the three modules yields super-additive synergistic performance gains. Individual modules only bring marginal metric improvements, while their integrated deployment achieves substantial performance uplifts. This verifies that the proposed architecture is a customized optimization scheme dedicated to lung medical images.

### 5.2. Clinical Application Value of the Model

Lung cancer ranks first worldwide in terms of morbidity and mortality among all malignant tumors. Timely detection of early micronodules can drastically raise patients’ 5-year survival rates. Nevertheless, clinical practice faces two prominent pain points: limited hardware resources in primary hospitals and high missed diagnosis rates during radiologists’ manual reading.

The lightweight model proposed in this work has a parameter count of merely 4.3 M and an inference speed of 141.3 FPS, enabling deployment on ordinary workstations and portable devices. Its recall rate reaches 87.9%, which greatly cuts missed detections of high-risk ground-glass nodules ranging from 3 mm to 5 mm and prevents delayed treatment for early lung cancer. The embedded CBAM suppresses false positives caused by vascular cross-sections, sparing patients from repeated CT follow-ups and unnecessary biopsies.

In generalization tests on the multi-center LIDC-IDRI dataset, the model exhibits negligible performance degradation and adapts to clinical CT images captured by diverse scanning devices. It holds great potential for large-scale screening across multiple medical centers and effectively eases the heavy workload of radiologists at primary care institutions.

### 5.3. Model Comparison

Compared with traditional machine learning algorithms based on handcrafted features, the proposed network eliminates manual texture feature engineering and achieves better adaptability to irregular small nodules.

In contrast to 3D convolution and two-stage high-precision detection models, our method delivers over sevenfold faster inference speed with only a slight drop in detection accuracy, greatly lowering hardware deployment requirements.

When benchmarked against modified YOLOv5/v7/v10 variants equipped with a single attention module, the three-tier collaborative optimization strategy in this work simultaneously addresses three core challenges: feature extraction, background noise interference, and regression of hard samples. It achieves far more prominent gains in recall for micronodules than existing lightweight alternatives. This design fills the performance gap between heavy high-precision detection models and low-accuracy lightweight baseline networks.

The proposed model only processes two-dimensional axial slices and fails to exploit three-dimensional spatial information across CT slices, leading to missed detections of elongated ground-glass nodules spanning multiple layers. Model training relies exclusively on publicly annotated datasets without validation against prospective multi-center clinical cohorts confirmed by pathological gold standards. Additionally, the absence of Grad-CAM visualization for interpretability analysis limits the clinical credibility of the detection outputs. Future research will incorporate lightweight three-dimensional dynamic convolutions to capture volumetric voxel features, expand the dataset with pathologically matched CT scans collected from multiple medical centers, integrate an auxiliary branch for nodule malignancy risk classification, and embed visualization modules to enhance model interpretability. Generative adversarial networks will also be adopted to synthesize additional samples of rare micronodules, ultimately refining an integrated computer-aided diagnosis system optimized for routine clinical deployment.

## 6. Conclusions

In this paper, we have presented an improved YOLOv8n algorithm for pulmonary nodule detection that addresses the critical trade-off between detection accuracy and computational efficiency. Through three synergistic modifications-the integration of ODConv in the backbone for adaptive feature extraction, multi-position CBAM attention embedding in the neck for noise suppression, and Wise-IoU loss for improved bounding box regression-we have achieved substantial performance improvements on the LUNA16 dataset. The proposed method improves Precision by 6.3%, Recall by 8.6%, mAP50 by 3.4%, and mAP50-95 by 2.7% compared to the original YOLOv8n, while maintaining an inference speed of 141.3 FPS. Stratified analysis confirms that the most significant improvements are achieved for the clinically critical category of small nodules (3–5 mm), where miss rates are traditionally highest.

Looking forward, several promising directions for future work can be identified. First, we plan to extend our 2D detection framework to incorporate 3D contextual information from adjacent CT slices, which should further improve detection performance for small and low-contrast nodules. Second, we will integrate a malignancy classification branch into the detection framework to provide more comprehensive clinical decision support. Third, we will conduct prospective validation on multi-center clinical datasets in collaboration with radiologists to evaluate the real-world performance and clinical utility of our system. Finally, we will explore more advanced lightweight architectures and knowledge distillation techniques to further optimize the model for deployment on edge computing devices. The lightweight, high-performance detection framework developed in this work represents an important step toward making accurate, real-time computer-aided diagnosis widely accessible in clinical practice.

## Figures and Tables

**Table 1:** Comparison of experimental results between YOLOv8 and attention mechanism.

Model	Precision%	Recall%	mAP50%	mAP50-95%	FPS
YOLOv8n	82.8	79.3	83.4	46.6	148.6
YOLOv8n + SE	81.7	79.9	82.1	46.2	140.3
YOLOv8n + SA	82.1	79.5	83.2	47.5	137.7
YOLOv8n + CA	81.5	80.2	82.6	47.9	134.5
YOLOv8n + CBAM	84.6	82.6	85.2	48.4	142.9

**Table 2:** Experimental results of adding attention mechanisms to different positions.

CBAM	Precision%	Recall%	mAP50%	mAP50-95%
Without CBAM	86.2	83.1	84.2	43.1
Only two CBAMs are embedded in upsampling layers	87.5	85.2	85.4	45.2
Only two CBAMs are embedded in downsampling layers	87.9	85.8	85.7	46
OURS	89.1	87.9	86.8	49.3

**Table 3:** Comparison of experimental results between YOLOv8 and loss function.

Model	Precision%	Recall%	mAP50%	mAP50-95%	FPS
YOLOv8n	82.8	79.3	83.4	46.6	148.6
YOLOv8n + EIoU	82.5	80.1	83.9	46.5	142.7
YOLOv8n + GIoU	81.4	79.6	82.6	47.0	146.3
YOLOv8n + SIoU	81.8	79.8	83.5	46.8	145.5
YOLOv8n + Wise-IoU	85.1	83.4	84.1	47.8	146.8

**Table 4:** Ablation Experiment.

Model	ODConv	CBAM	Wise-IoU	Precision%	Recall%	mAP50%	mAP50-95%
YOLOv8n				82.8	79.3	83.4	46.6
1	√			85.5	81.1	85.1	48.1
2		√		84.6	82.6	85.2	48.4
3			√	85.1	83.4	84.1	47.8
4	√	√		87.8	84.9	86.4	48.8
5	√		√	87.2	85.6	85.7	48.6
6		√	√	86.6	85.8	84.6	47.9
7	√	√	√	89.1	87.9	86.8	49.3

**Table 5:** Detection Metrics of the Model on the LUNA16 and LIDC-IDRI Datasets.

Datasets	Model	Precision%	Recall%	mAP50%	FPS
LUNA16	Baseline YOLOv8n	82.8	79.3	83.4	148.6
LUNA16	Proposed Model	89.1	87.9	86.8	141.3
LIDC-IDRI	Baseline YOLOv8n	81.5	77.6	82.1	147.9
LIDC-IDRI	Proposed Model	87.4	86.2	85.3	139.7

**Table 6:** Model Comparison Results.

Model	Precision %	Recall %	mAP50 %	mAP50-95%	FPS	Parameter (M)
YOLOv8n	82.8	79.3	83.4	46.6	148.6	3.2
YOLOv5n	79.8	78.6	83.2	43.5	142.3	3.6
YOLOv7n	76.4	73.6	80.1	42.8	102.3	9.77
YOLOv9s	83.9	82.0	85.7	46.8	70.4	14.2
YOLOv10n	84.2	82.3	85.9	47.1	126.5	4.8
Yolov11m	81.2	79.7	83.9	49.6	116	20.1
Faster-RCNN	83.2	82.1	85.4	44.8	91.1	27.4
SSD	83.8	80.8	84.6	41.3	97.7	10.2
3D Faster RCNN	90.2	88.7	88.7	50.6	18.6	62.3
OURS	89.1	87.9	86.8	49.3	141.3	4.3

## Data Availability

The LUNA16 dataset supporting the findings of this study is available at https://luna16.grand-challenge.org/ (accessed on 6 June 2026), and the LIDC-IDRI dataset can be obtained via https://wiki.cancerimagingarchive.net/pages/viewpage.action?pageId=1966254 (accessed on 6 June 2026). The standardized preprocessing code and data division rules used in this paper are available from the author upon reasonable request.

## Abbreviations

The following abbreviations are used in this manuscript:

ODConv	Omni-dimensional Dynamic Convolution
CBAM	Convolutional Block Attention Module
CT	Computerized tomography
CAD	Computer-aided Diagnosis
CNN	Convolutional neural network
C2f	Cross-Stage Partial bottleneck with two convolutions
FPN	Feature Pyramid Network
PAN	Path Aggregation Network
LUNA16	Lung Nodule Analysis 2016
LIDC-IDRI	Lung Image Database Consortium and Image Database Resource Initiative
WIoU	Wise Intersection over Union
TP	True Positive
FP	False Positive
FN	False Negative
FPS	Frames per second
FROC	Free-Response Receiver Operating Characteristic

## References

[1] Bray F., Laversanne M., Sung H., Ferlay J., Siegel R.L., Soerjomataram I., Jemal A. (2024). Global Cancer Statistics 2022: GLOBOCAN Estimates of Incidence and Mortality Worldwide for 36 Cancers in 185 Countries. CA A Cancer J. Clin..

[2] Liu W., Wu Y., Zheng Z., Bittle M., Yu W., Kharrazi H. (2025). Enhancing Diagnostic Accuracy of Lung Nodules in Chest Computed Tomography Using Artificial Intelligence: Retrospective Analysis. J. Med. Internet Res..

[3] Yang F., Wei G., Cao H., Xing M., Liu J., Zhang J. (2020). Research Progress on Content-Based Medical Image Retrieval. Laser Optoelectron. Prog..

[4] Liu X., Qi S., Xiong P., Wang Y. (2020). An Automatic Detection Algorithm for Pulmonary Nodules That Integrates Multi-Scale Information. J. Biomed. Eng..

[5] Czerw A., Deptała A., Partyka O., Pajewska M., Wiśniewska E., Sygit K., Wysocki S., Cipora E., Konieczny M., Banaś T. (2024). Lung Cancer Screening—Trends and Current Studies. Cancers.

[6] Mohamed E., Fletcher D., Hart S., Guinn B. (2024). Can Tumour Antigens Act as Biomarkers for the Early Detection of Non-Small Cell Lung Cancer?. Onco.

[7] Peters A.A., Wiescholek N., Müller M., Klaus J., Strodka F., Macek A., Primetis E., Drakopulos D., Huber A.T., Obmann V.C. (2024). Impact of Artificial Intelligence Assistance on Pulmonary Nodule Detection and Localization in Chest CT: A Comparative Study among Radiologists of Varying Experience Levels. Sci. Rep..

[8] Kermany D.S., Goldbaum M., Cai W., Valentim C.C.S., Liang H., Baxter S.L., McKeown A., Yang G., Wu X., Yan F. (2018). Identifying Medical Diagnoses and Treatable Diseases by Image-Based Deep Learning. Cell.

[9] Hsu H.-H., Ko K.-H., Chou Y.-C., Wu Y.-C., Chiu S.-H., Chang C.-K., Chang W.-C. (2021). Performance and Reading Time of Lung Nodule Identification on Multidetector CT with or without an Artificial Intelligence-Powered Computer-Aided Detection System. Clin. Radiol..

[10] Marinakis I., Karampidis K., Papadourakis G. (2024). Pulmonary Nodule Detection, Segmentation and Classification Using Deep Learning: A Comprehensive Literature Review. BioMedInformatics.

[11] Kim J. (2024). Studies and Real-World Experience Regarding the Clinical Application of Artificial Intelligence Software for Lung Nodule Detection. J. Korean Soc. Radiol..

[12] Chen J., Cao R., Jiao S., Dong Y., Wang Z., Zhu H., Luo Q., Zhang L., Wang H., Yin X. (2023). Application Value of a Computer-Aided Diagnosis and Management System for the Detection of Lung Nodules. Quant. Imaging Med. Surg..

[13] Xiong Y., Deng L., Wang Y. (2024). Pulmonary Nodule Detection Based on Model Fusion and Adaptive False Positive Reduction. Expert Syst. Appl..

[14] Liu Z., Shi Y., Lin Y., Yang Y. (2023). Intelligent Medicine and Beyond. Chin. Sci. Bull..

[15] Gao C., Wu L., Wu W., Huang Y., Wang X., Sun Z., Xu M., Gao C. (2024). Deep Learning in Pulmonary Nodule Detection and Segmentation: A Systematic Review. Eur. Radiol..

[16] Akram S., Javed M.Y., Hussain A., Riaz F., Usman Akram M. (2015). Intensity-Based Statistical Features for Classification of Lungs CT Scan Nodules Using Artificial Intelligence Techniques. J. Exp. Theor. Artif. Intell..

[17] Yin S., Li H., Teng L. (2020). Airport Detection Based on Improved Faster RCNN in Large Scale Remote Sensing Images. Sens. Imaging.

[18] Li W. (2021). Analysis of Object Detection Performance Based on Faster R-CNN. J. Phys. Conf. Ser..

[19] Sun L., Wang Z., Pu H., Yuan G., Guo L., Pu T., Peng Z. (2021). Attention-Embedded Complementary-Stream CNN for False Positive Reduction in Pulmonary Nodule Detection. Comput. Biol. Med..

[20] Tao G., Zhou F., Liu F., Gui H., Luo F., Huang W.C., Ni Zou J., Phoebe Chen Y.P. (2023). DFNodule: A Novel Deformable Faster R-CNN for Lung Nodule Detection. Proceedings of the 2023 IEEE International Conference on Bioinformatics and Biomedicine (BIBM).

[21] Zhou D., Xu H., Liu W., Liu F. (2025). LN-DETR: Cross-Scale Feature Fusion and Re-Weighting for Lung Nodule Detection. Sci. Rep..

[22] Terven J., Córdova-Esparza D.-M., Romero-González J.-A. (2023). A Comprehensive Review of YOLO Architectures in Computer Vision: From YOLOv1 to YOLOv8 and YOLO-NAS. Mach. Learn. Knowl. Extr..

[23] Zhao K., Qin H., Li X., Zhang Y. (2024). Real-Time Pulmonary Nodule Detection Algorithm Combining Attention and Multipath Fusion. J. Comput. Appl..

[24] Setio A.A.A., Traverso A., De Bel T., Berens M.S.N., Bogaard C.V.D., Cerello P., Chen H., Dou Q., Fantacci M.E., Geurts B. (2017). Validation, Comparison, and Combination of Algorithms for Automatic Detection of Pulmonary Nodules in Computed Tomography Images: The LUNA16 Challenge. Med. Image Anal..

[25] Hussain M. (2023). YOLO-v1 to YOLO-v8, the Rise of YOLO and Its Complementary Nature toward Digital Manufacturing and Industrial Defect Detection. Machines.

[26] Li C., Zhou A., Yao A. (2022). Omni-Dimensional Dynamic Convolution. arXiv.

[27] Woo S., Park J., Lee J.-Y., Kweon I.S., Ferrari V., Hebert M., Sminchisescu C., Weiss Y. (2018). CBAM: Convolutional Block Attention Module. Computer Vision—ECCV 2018.

[28] Tong Z., Chen Y., Xu Z., Yu R. (2023). Wise-IoU: Bounding Box Regression Loss with Dynamic Focusing Mechanism. arXiv.

[29] Yang L., Honarvar Shakibaei Asli B. (2025). MSConv-YOLO: An Improved Small Target Detection Algorithm Based on YOLOv8. J. Imaging.

[30] Park D. (2024). A Comprehensive Review of Performance Metrics for Computer-Aided Detection Systems. Bioengineering.

